# Testing the binomial fixed effects logit model, with an application to female labour supply

**DOI:** 10.1007/s00181-021-02034-2

**Published:** 2021-03-06

**Authors:** Rainer Winkelmann, Lin Xu

**Affiliations:** Department of Economics, University of Zurich, Zurich, Switzerland

**Keywords:** Proportions data, Unobserved heterogeneity, Conditional maximum likelihood, Overdispersion, C23, J21

## Abstract

Regression models for proportions are frequently encountered in applied work. The conditional expectation function is bounded between 0 and 1 and therefore must be nonlinear, requiring nonstandard panel data extensions. One possible approach is the binomial panel logit model with fixed effects (Machado in J Econom 119:73–98, 2004). We propose a new and simple implementation of this conditional maximum likelihood estimator for standard software. We investigate the properties of the estimator under misspecification and derive a new test for overdispersion. Estimator and test are applied in a study of contracted working volumes, measured as proportion of full-time work, for women in Switzerland.

## Introduction

After half a century of research on econometric models for limited dependent variables (Maddala 1983; Wooldridge 2002), it remains the case that only a small portion of it deals with proportions data, and even a smaller one with panel models for such proportions. Machado (2004) proposes the binomial fixed effects logit model, Papke and Wooldridge (2008) a correlated random effects probit quasi-likelihood estimator, and Ramalho et al. (2016) a class of exponential GMM estimators.

And yet, proportions and related types of data are regularly encountered in applied econometric work. They sometimes correspond to the fraction of “successes” in a sequence of Bernoulli trials. Examples are the proportion of successful patent applications (Machado 2004) and the proportion of days absent from work (Barmby et al. 2001). Similarly, variety scores (e.g. the number of applicable items in a general health questionnaire), bounded count data, as well as ratings, can be re-scaled to the (0, 1)-interval. For example, the degree of (customer or life) satisfaction in surveys often has a lower bound of zero (meaning “completely dissatisfied”) and some upper bound (e.g. 10, meaning “completely satisfied”), which can then be re-coded as 100%. All these variables share the key features of being discrete and bounded, and the binomial model with a logit function for the expected proportion provides a natural starting point for modelling.

For the fixed effects setting, Machado (2004) shows that the incidental parameters problem can be overcome by a conditional maximum likelihood (CML) estimator, much like it is the case for the binary response logit model (Chamberlain 1980). She also provides Monte Carlo evidence indicating that the dummy variables (DV) approach is subject to an upward bias that is decreasing both in the length of the panel, *T*, and in the number of Bernoulli trials, *K*. For $T > 5$ and $K > 5$, CML and DV approaches yield quite comparable results with minor bias (Machado 2004).

This paper advances the earlier literature in three directions: First, we show how the binomial logit fixed effects estimator can be implemented in any off-the-shelf statistical software that includes a conditional logit routine, using the idea of cloning, or data expansion. Second, we study the properties of the CML and DV estimators for the case where the binomial distributional assumption fails. The leading example is that of overdispersion, originating from random unobserved heterogeneity or dependence among the Bernoulli trials. The CML estimator is not a pseudo-ML estimator in the sense of Gourieroux et al. (1984), and it does not possess formal robustness properties. We therefore investigate the extent of bias in a series of simulation experiments. Third and finally, we derive and implement a new test for the binomial assumption, i.e. a test for the hypothesis of no dispersion, as existing tests (e.g. Dean 1992) cannot be applied because the fixed effects are not estimated by the CML estimator.

To illustrate the proposed methods, we conduct a study of the determinants of women’s work behaviour in Switzerland. The outcome variable is the contracted work-time percentage, where 0 means no work and 1 means full-time work. Data are extracted from the Swiss Household Panel for the years 2012–2016. The binomial logit estimates indicate that having children is associated with substantially reduced work-time percentage, ceteris paribus. Perhaps more surprisingly, having a partner makes the effect more pronounced, whereas speaking French reduces it.

## Model and estimation

A proper panel model for proportions $y_{it} \in [0,1]$ must overcome two challenges. First, the model should observe the restricted support of the outcome, as well as being able to handle data clustering at the end points. For instance, the log-odds transformation $\log [y_{it}/(1-y_{it})]$ is not defined for $y_{it} =0$ or $y_{it} =1$. Another method facing the same limitation is beta regression, which is flexible for fitting continuous proportional data but cannot give predictions at the boundaries with positive probability. Second, direct control for unobserved time-invariant individual heterogeneity (that may or may not be correlated with the regressors), using a dummy for each cross-sectional unit is subject to the incidental parameters problem, leading to inconsistent estimation of structural parameters when the length of panel *T* is fixed.

Machado (2004) addresses these two issues by considering a binomial logit model with fixed effects. The application she had in mind was using information on the number of patent applications and patents granted at the firm level to estimate the probability of obtaining a patent (i.e. proportion of patents granted). She derived a consistent conditional maximum likelihood estimator based on the following assumptions:

### Assumption 1

Let $Y_{it} = K y_{it}$, where *K* is a known integer and

$$\begin{aligned} y_{it} \in \left\{ 0, \frac{1}{K }, \frac{2}{K },\ldots ,1 \right\} \end{aligned}$$

such that

1 $$\begin{aligned} Y_{it} |p_{it} \sim \text {binomial} (K , p_{it}) \,,\qquad i = 1,\ldots ,N; \qquad t = 1,\ldots ,T \end{aligned}$$

Here, *K* is the number of “trials”, $Y_{it} = K y_{it}$ is the “number of successes”, and $y_{it}$ is the proportion, or fraction of successes for observation unit *i* in period *t*.

### Assumption 2

Let the expected proportion depend on covariates $x_{it}$, and an individual-specific effect $\alpha _i$ as follows:

2 $$\begin{aligned} E(y_{it}|x_{it}, \alpha _i) = p_{it} = \frac{\exp (x_{it}'\beta + \alpha _i)}{1+ \exp (x_{it}'\beta + \alpha _i)} \equiv \Lambda _{it} \end{aligned}$$

$x_{it}$ and $\alpha _i$ can be correlated.

### Assumption 3

Observations are independent between individuals and, conditional on group effects $\alpha _i$, serially uncorrelated.

The objective of the analysis is estimation of $\beta$. Under Assumptions 1–3, the joint binomial density for $Y_{i1},Y_{i2},\ldots ,Y_{iT}$ conditional on $\sum _t Y_{it}$ is given by (see Machado 2004)

3 $$\begin{aligned} f\left( Y_{i1},Y_{i2},\ldots ,Y_{iT}|\sum _t Y_{it}\right)= & {} \frac{\Pi _t {K \atopwithdelims ()Y_{it}} p_{it}^{Y_{it}} (1-p_{it})^{K-{Y_{it}}}}{\sum _{q \in Q_i}\Pi _t {K \atopwithdelims ()q_{t}} p_{it}^{q_t} (1-p_{it})^{K-{q_t}}} \nonumber \\= & {} \frac{\exp (\sum _t Y_{it} x_{it}'\beta ) \Pi _t {K \atopwithdelims ()Y_{it}} }{\sum _{q \in Q_i} \exp (\sum _t q_{t} x_{it}'\beta ) \Pi _t {K \atopwithdelims ()q_{t}}} \end{aligned}$$

where $Q_i=\{(q_1,q_2,\ldots ,q_T)|q_t \in \{0,1,2,\ldots ,K \}, \sum _t q_t=\sum _t Y_{it} \}$. The conditional binomial approach eliminates the fixed effects $\alpha _i$ which appear in the numerator and denominator with same power. Observations for which $\sum _t Y_{it}=0$ or $\sum _t Y_{it}=KT$ have a conditional probability of 1 and do not contribute to estimation of $\beta$. For proportion data, such outcomes tend to be much less prevalent than they are for binary outcomes.

In principle, the Machado (2004) approach solves an important problem in the analysis of panel data for proportions. In contrast to Papke and Wooldridge (2008), it is “semi-parametric”, as there is no need to specify the relationship between the individual effect and the regressors, and also no need to add an assumption on the distribution of the individual effects. And yet, subsequent applications have been few, perhaps, because the estimator has a couple of limitations. First, the estimator is not readily available in standard econometric software packages. We therefore develop a simple modification that makes it easily implementable in standard software. And second, the binomial assumption may be violated, and the properties of the estimator under misspecification are unknown so far. We provide such a misspecification analysis in Sect. 2.3, and also derive a test for the binomial assumption in Sect. 3. In addition, it is important to point out that the binomial fixed effects estimator can be applied in a broader range of situations than hitherto considered, i.e. beyond those relating to the number (or proportion) of successes in a sequence of *K* independent Bernoulli trials. Even in the absence of such a process, the model can be a good starting point for fractions and shares, as we illustrate in an application to work-time percentages.

### An alternative implementation

To understand, how the binomial logit fixed effects estimator can be implemented using any off-the-shelf statistical software with a conditional logit routine, note that the binomial distribution arises as the sum of *K* independent Bernoulli trials. Therefore, two estimators are equivalent: one based on a binomial log-likelihood function and the other based on a Bernoulli log-likelihood for an expanded dataset.

For the expanded dataset, one simply generates a sequence of *K* copies for each *i*, keeping the regressors unchanged, where the proportion $y_{it}$ is replaced by a sequence of 0/1 indicator variables $d_{ijt}$ in arbitrary order such that

4 $$\begin{aligned} \sum _{j=1}^K d_{ijt} = K y_{it} \end{aligned}$$

It follows that $d_{ijt}$ and $y_{it}$ have the same CEF:

5 $$\begin{aligned} E(y_{it}|x_{it})= E\left( \left. \frac{\sum _{j=1}^K d_{ijt}}{K} \right| x_{it} \right) = E(d_{ijt}|x_{it}). \end{aligned}$$

The logit (Bernoulli) log-likelihood function of the expanded dataset is given by

6 $$\begin{aligned} \log L= & {} \sum _t^{T} \sum _i^{N} \left[ \sum _j^{K} d_{ijt}\log (\Lambda _{it})+(1-d_{ijt})\log (1-\Lambda _{it}) \right] \nonumber \\= & {} \sum _t^{T} \sum _i^{N} Y_{it} \log (\Lambda _{it})+ (K-Y_{it}) \log (1-\Lambda _{it}). \end{aligned}$$

This log-likelihood function is equal to the binomial log-likelihood as well as to the Bernoulli quasi-log-likelihood (Papke and Wooldridge 1996, replacing $Y_{it}$ by $y_{it}$ and $(K-Y_{it})$ by $(1-y_{it})$), up to an additive constant, and the three ML estimators are therefore identical.

Similarly, the conditional density function for individual *i* at time *t* can be written as:

7 $$\begin{aligned} f \left( \{ d_{ijt} \}|\sum _t \sum _j d_{ijt}\right) =\frac{ \Pi _t \Pi _j p_{it}^{d_{ijt}} (1-p_{it})^{1-d_{ijt}}}{\sum _{s \in S_i}\Pi _t \Pi _j p_{it}^{s_{jt}} (1-p_{it})^{1-{s_{jt}}}} =\frac{\exp \left( \sum _t \sum _j d_{ijt} x_{it}'\beta \right) }{\sum _{s \in S_i} \exp \left( \sum _t \sum _j s_{jt} x_{it}'\beta \right) } \end{aligned}$$

where $$S_i=\{ (s_{11},s_{21},\ldots ,s_{K1},s_{12},\ldots , s_{KT})|s_{jt} \in \{ 0,1 \}, \sum _t \sum _j s_{jt}=\sum _t \sum _j d_{ijt}) \}$$.

Compared with Eq. (3), the number of *s* such that $\{s|\sum _j s_{ijt}= q_{it}\}$ is ${K \atopwithdelims ()q_{it}}$ for given *q*. Equation (7) is therefore basically the same as Eq. (3), except for the term $\Pi _t {K \atopwithdelims ()Y_{it}}$ in the numerator of (3). But this term does not depend on any parameter and thus does not affect the first-order condition for the maximum of the log-likelihood function. Specifically, the conditional Bernoulli log-likelihood function is given by:

8 $$\begin{aligned} \log L=\sum _i \left[ \sum _t \sum _j d_{ijt} x'_{it}\beta -\log \left( \sum _{s \in S_i} \exp \left( \sum _t \sum _j s_{jt} x_{it}'\beta \right) \right) \right] \end{aligned}$$

with the first derivative

9 $$\begin{aligned} \frac{\partial \log L}{\partial \beta }=\sum _i\left[ \sum _t Ky_{it} x'_{it} -\frac{\sum _{s \in S_i} \exp \left( \sum _t \sum _j s_{jt} x_{it}'\beta \right) \sum _t \sum _j s_{jt}x'_{it}}{\sum _{s \in S_i} \exp \left( \sum _t \sum _j s_{jt} x_{it}'\beta \right) }\right] \end{aligned}$$

which is the same as that of the conditional binomial model and therefore will yield the same consistent estimator of $\beta$, after elimination of the fixed effects. We from now on refer to this estimator as the Blogit (for binomial logit) conditional maximum likelihood estimator, or in short, Blogit CML, in contrast to the inconsistent binomial estimator with dummy variables included for each cross-sectional unit, Blogit DV.

Of course, there can be situations where the expansion approach becomes practically infeasible: as *K* gets large, for instance, because proportions are measured at the granularity of percentage points, the size of the set $S_i$ of the conditional Bernoulli log-likelihood expressions is increased from *T* to $100\times T$, at which point one may run into computational constraints.

### Overdispersion

Departures from the binomial proportions model can take a number of forms. The first one is a violation of the independence assumption for the underlying Bernoulli trials. Positive dependence, or contagion, among the sequence of Bernoulli trials causes overdispersion, a conditional variance exceeding the binomial variance $K p_{it}(1-p_{it})$. Another violation stems from “random unobserved heterogeneity”. This is in addition to the time-invariant unobserved heterogeneity $\alpha _i$. Random unobserved heterogeneity is time- and individual-specific, as well as unrelated to $x_{it}$. Specifically, it means that $p_{it}$ is no longer a constant but rather a random variable, say ${\tilde{p}}_{it}$. Marginalizing over ${\tilde{p}}_{it}$ then leads to a mixture model that is characterized by overdispersion as well. Depending on the distribution of $\tilde{p}_{it}$, proportions can, for example, have a u-shaped probability function even conditional on $\alpha _i$ and $x_{it}$, i.e. probability mass stacked at the endpoints of 0 and 1, which is never the case for a binomial distribution that has either a single, or two adjacent modes.

A prominent example for a continuous mixture is the beta-binomial model, where the conditional probability is

10 $$\begin{aligned} {\tilde{p}}_{it} \sim \mathrm{beta}(u_{it},v_{it}), \end{aligned}$$

and

$$\begin{aligned} u_{it}=\phi \Lambda (x_{it}'\beta +\alpha _i), v_{it}=\phi (1-\Lambda (x_{it}'\beta +\alpha _i)). \end{aligned}$$

where $\phi >0$ is a parameter that determines the degree of overdispersion. It is straightforward to show that a beta-binomial distribution with this parameterization has expectation $K \Lambda _{it}$ and variance

11 $$\begin{aligned} \mathrm{Var}(Y_{it}|K, \Lambda _{it},\phi )= K \Lambda _{it}(1-\Lambda _{it})\left( 1+\frac{K-1}{\phi +1}\right) \end{aligned}$$

Thus, the variance of the beta-binomial model is proportional to that of the binomial model. Overdispersion increases in *K*, the number of trials, and it decreases in the parameter $\phi$. The binomial variance is obtained for $K=1$, or in the limit, for $\phi \rightarrow \infty$, which also means that $\text{ Var }({\tilde{p}}_{it}) \rightarrow 0$.

In general, fixed effects conditional maximum likelihood estimators are not consistent if the underlying model is misspecified. The reason is that the first-order condition is not a moment condition for the mean, but rather a function of the conditional probabilities. However, it might still be the case that the CML estimator works satisfactorily as long as the degree of overdispersion, in other words, the departure from the binomial assumption, is not too large. We will explore this type of robustness in a series of simulation experiments. We thereby extend results by Machado (2004), who considered the severity of the incidental parameters problem and the small sample properties of the CML estimator under the maintained assumption of a correctly specified binomial model. In our simulations, this assumption is dropped.

### Simulation study

The simulation experiments employ two different data generating processes: one where the binomial assumption is satisfied, and the other, based on the beta-binomial model, where overdispersion is present. Unobserved time-invariant individual heterogeneity is positively correlated with the regressor in both cases. The degree of overdispersion is varied from 10 to 200%.

Both set-ups use the same logit conditional expectation function with a single regressor

12 $$\begin{aligned} E(y_{it}|x_{it},\alpha _i)= \Lambda (\beta _0 + \beta _1 x_{it} +\alpha _i)= \frac{\exp (\beta _0 + \beta _1 x_{it}+\alpha _i)}{1+\exp (\beta _0 + \beta _1 x_{it}+\alpha _i)} \qquad , \end{aligned}$$

where $\beta _0=0$, $\beta _1=2$ and the size of the cross section is either $N=100$ or $N=500$. The time dimension increases from $T=2$, $T=5$ to $T=10$.

The regressor $x_{it}$ is drawn from a uniform distribution with support $[-1,1]$ and has therefore a mean of 0 and a variance of 1/3. Draws are independent both across individuals and over time. We make a correlated random effects assumption:

13 $$\begin{aligned} \alpha _i= \sqrt{T} {\bar{x}}_i + \varepsilon _i, \end{aligned}$$

where $\varepsilon _i\sim N(0,1)$. It follows that the correlation between $\alpha _i$ and ${\bar{x}}_i$ is 0.5, a substantial amount.

Once the mean is given, the dependent variable is obtained by generating pseudo-random numbers from either a binomial or a beta-binomial distribution. Specifically, we first draw integer random numbers from a (beta) binomial distribution with parameters *K* and $\Lambda (x_{it}\beta _{1}+\alpha _i)$ and then divide the result by the number of categories *K*, e.g.

14 $$\begin{aligned} y_{it}=\frac{K y_{it}}{K}, K y_{it} \sim \mathrm{binomial}(K, p_{it}),p_{it}= \Lambda (\beta _0 + \beta _1 x_{it}+\alpha _i) \end{aligned}$$

*K* is exogenously set to 2, 5 or 10. For $K=2$, $K \times y_{it}$ can be 0, 1, or 2, with corresponding fractions of $y_{it} = 0, 0.5$, or 1, respectively; if $K=10$, $y_{it}$ takes on one-digit decimals: 0, 0.1, 0.2,..., 1.

Ignoring the presence of the individual-specific component and estimating the marginal, pooled model instead has two effects:

- $\beta _1$ is upward biased due to the positive correlation between $x_{it}$ and $\alpha _i$.
- $\beta _1$ is downward biased due to omitted heterogeneity. In the probit model, there is a closed form expression for this bias (Wooldridge 2002). In the logit model, it needs to be computed numerically, but the direction is the same.

Which one of the two biases is larger is an empirical matter. The DV estimator, on the other hand, suffers from the standard incidental parameters bias that is upward (Abrevaya 1997).

Table 1 shows the simulation results based on 1000 replications, for a sample size of $N=100$. The mean and standard deviation of estimated coefficients across replications are reported. Three estimators were used: Blogit CML, Blogit DV, and pooled logit, respectively. Similar to Machado (2004), we find that the Blogit CML model estimates the true structural slope parameter very well even for small samples. There is a 2% upward bias for $T=K=2$ that vanishes quickly as either *T* or *K* increases. The sampling variability decreases not only in *T* but also in *K*, albeit at a less than $\sqrt{K}$ rate. The Blogit DV estimator has a larger bias and a larger standard error, and hence a higher mean squared error, in all settings. The bias becomes small as *T* and *K* increase. For instance, for $T=10$ and $K=10$, the mean Blogit DV estimate is 2.025, whereas the mean Blogit CML estimate is 2.000. On the other hand, the pooled logit estimator has no tendency to converge to the true parameter $\beta _1$ = 2, over- or underestimating it depending on *K* and *T*. In the lower panel of Table 1, simulations are repeated for a larger sample, with $N=500$ instead of $N=100$. The qualitative conclusions remain unchanged.

**Table 1:** Simulation results under the binomial distribution

$N=100$	$T=2$			$T=5$			$T=10$		
	Blogit CML	Blogit DV	Pooled logit	Blogit CML	Blogit DV	Pooled logit	Blogit CML	Blogit DV	Pooled logit
$K=2$	2.049	2.880	2.242	2.003	2.280	1.986	2.006	2.134	1.877
	(0.419)	(0.621)	(0.255)	(0.178)	(0.211)	(0.145)	(0.118)	(0.128)	(0.101)
$K=5$	2.012	2.279	2.243	2.000	2.101	1.990	2.002	2.051	1.872
	(0.233)	(0.272)	(0.190)	(0.111)	(0.118 )	(0.104)	(0.073)	(0.075)	(0.076)
$K=10$	2.005	2.129	2.241	2.001	2.050	1.989	2.000	2.025	1.871
	(0.157)	(0.169)	(0.153)	(0.078)	(0.081)	(0.091)	(0.052)	(0.052)	(0.063)
$$N=500$$									
$K=2$	2.013	2.826	2.234	2.002	2.278	1.987	2.002	2.130	1.870
	(0.170)	(0.254)	(0.112)	(0.082)	(0.097)	(0.068)	(0.053)	(0.057)	(0.046)
$K=5$	2.003	2.268	2.232	2.001	2.102	1.986	2.000	2.049	1.867
	(0.102)	(0.119)	(0.078)	(0.051)	(0.054)	(0.050)	(0.033)	(0.034)	(0.033)
$K=10$	1.997	2.121	2.235	1.999	2.049	1.984	2.000	2.024	1.867
	(0.071)	(0.076)	(0.067)	(0.034)	(0.035)	(0.042)	(0.023)	(0.023)	(0.029)

Results for 1000 Monte Carlo replications; standard deviations in parentheses. The number of observation in each period is 100 in the upper part of the table and 500 in the second half. $x_{it} \sim U[-1,1]$. $\beta _1= 2$ and $\alpha _i = \sqrt{T} \bar{x}_i + N(0,1)$. Outcome $y_{it}$ follows the binomial distribution with $p_{it}= \frac{exp(x_{it}\beta _1+ \alpha _i)}{1+exp(x_{it}\beta _1+ \alpha _i)}$

*Blogit CML* denotes the binomial logit conditional maximum likelihood estimator; *Blogit DV* is the binomial logit fixed effects estimator including dummy variables for each individual; and *Pooled Logit* is the pooled logit estimator that ignores the presence of individual effects

### Beta-binomial DGP

Simulations from the beta-binomial model add a further step: instead of directly obtaining binomial responses with (conditional on $x_{it}$ and $\alpha _i$) success probability $p_{it}= \Lambda (\beta _0 + \beta _1 x_{it} +\alpha _i)$, ${\tilde{p}}_{it}$ is now drawn from a beta distribution with mean $p_{it}$:

15 $$\begin{aligned} {\tilde{p}}_{it} \sim \mathrm{beta}(\phi \Lambda (\beta _0 + \beta _1 x_{it}+\alpha _i), \phi (1-\Lambda (\beta _0 + \beta _1 x_{it}+\alpha _i))) \end{aligned}$$

From (11), we know that the multiplicative variance inflation factor depends both on *K* and $\phi$. To keep the degree of overdispersion constant for $K=2,5,10$, we adjust $\phi$ accordingly. For example, for 10% overdispersion and $K=2$, we have $1+(K-1)/(\phi +1)=1.1$, so $\phi = 9$.

As a practical limitation, common beta random number generators set lower bounds (above the theoretical ones of 0) for the two parameters. In Stata, for example, these are given by 0.05 and 0.15, respectively. From (15) we see that attempts to draw from the beta using arguments violating these bounds are more likely to arise when the mean is close to zero or one, or when $\phi$ is small (and therefore the degree of overdispersion is large). Since such occurrences only depend on exogenous factors, dropping these cases does not invalidate the estimation procedure. However, it affects the effective sample size and thus leads to higher standard errors than would otherwise be the case.

**Fig. 1:**
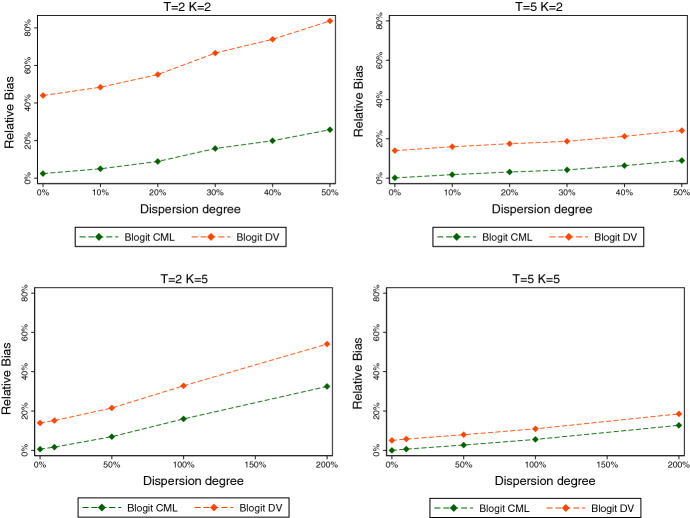
Relative bias by dispersion degree $(N=100)$

**Fig. 2:**
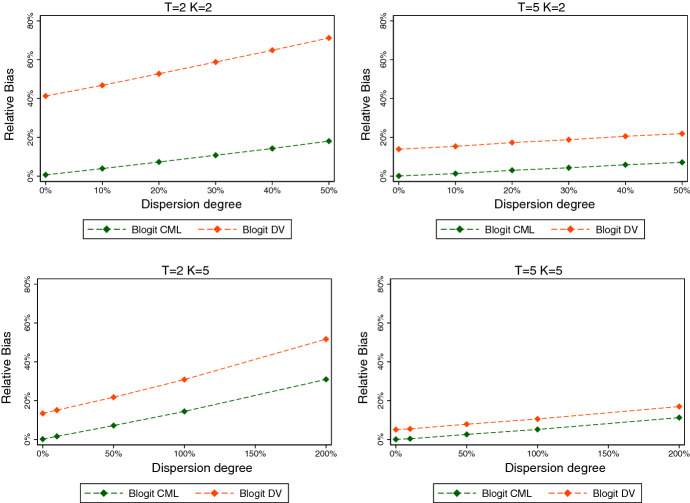
Relative bias by dispersion degree $(N=500)$

Figures 1 and 2 plot the relative biases of Blogit CML and Blogit DV against the degree of overdispersion, for $N=100$ and $N=500$, respectively. Overdispersion varies from 10% to 200%. (The full results on the means and standard deviations of the estimators for each DGP are given in Tables 6 and 7 in “Appendix.”)

Three key patterns emerge. First, overdispersion leads to an upward bias of both the Blogit CML and the Blogit DV estimators. The bias increases in the amount of overdispersion. Second, the Blogit CML estimator always dominates the Blogit DV estimator, both in terms of bias and standard error. The same pattern was already found for the binomial case, and it persists in the presence of overdispersion. Third, for a given degree of overdispersion, the bias is decreasing in *T* as well as in *K*. However, increasing *K* alone not necessarily leads to a reduction in estimation bias, because it increases the amount of overdispersion, ceteris paribus. Again, results are qualitatively similar for $N=500$ (see Fig. 2).

The overall conclusion is that the Blogit CML estimator maintains a rather good performance even if the binomial model is misspecified, as long as the degree of overdispersion is modest, or else, as long as *T* is large. To take the two extreme cases, for $N=100$ and $K=T=2$, the mean estimate with 10% overdispersion is 2.1, a 5% upward bias. For $K=T=10$, the mean estimate with 100% overdispersion is 2.049, a 2.45% upward bias.

## A test for overdispersion

Existing binomial tests for $y_{it}$, e.g. Dean’s (1992) score test or regression-based tests regressing squared residuals $y_{it} - {{\hat{\Lambda }}}_{it}$ on ${{\hat{\Lambda }}}_{it} (1-{{\hat{\Lambda }}}_{it})$, require estimates ${\hat{\Lambda }}_{it}$ in order to obtain conditional variances ${\widehat{\text{ Var }}}(y_{it}|x_{it})$. However, the Blogit CML approach does not give us ${\hat{\alpha }}_i$, so this is not feasible. To ascertain the validity of the Blogit CML model assumption, i.e. that $Ky_{it}$ is binomial distributed conditional on $\alpha _i$ and $x_{it}$, we propose an alternative approach that uses ${\hat{\beta }}$ but does not require estimates of $\alpha _i$, based on taking differences.

To start, consider a binary random variable $M_{it}$ defined by a draw from a Bernoulli distribution with mean $y_{it}$, $M_{it} \sim \mathrm{Bernoulli}(y_{it})$. Clearly, the conditional mean is $E(M_{it}|y_{it}) = y_{it}$, while the unconditional mean is $E(M_{it}) = \Lambda _{it}$. The conditional variance is $\text{ Var }(M_{it}|y_{it}) = y_{it}(1-y_{it})$, while the unconditional variance is $\text{ Var }(M_{it}) =E[y_{it}(1-y_{it})] + \text{ Var }(y_{it})= \Lambda _{it}(1-\Lambda _{it})$.

The basic idea of the test is to compare the variances of the differences $Y_{it}-Y_{is}$ and that of the difference $M_{it}-M_{is}$, for pairs of observations where the underlying probabilities $p_{it} = \Lambda _{it}$ are the same (or similar) for the two periods. For notational simplicity, let $t=1$ and $s=2$. In such cases, outcomes $Y_{i1}$, $Y_{i2}$ can be regarded under $H_0$ as random draws from i.i.d. binomial distributions and the variance of $Y_{i1}- Y_{i2}$ should be equal to the sum of binomial variances, under assumptions A1 and A3. On the other hand, the Bernoulli draws from the same distributions have standard variances. If there is over- or under-dispersion, the variance of $Y_{i1}-Y_{i2}$ will be larger or smaller than the variance calculated from Bernoulli draws.

Specifically, consider the variable

16 $$\begin{aligned} z_i= \frac{(Y_{i1}-Y_{i2})^2- K(M_{i1}- M_{i2})^2}{K(K-1)}. \end{aligned}$$

Conditional on $$y_{i1}$$, $$y_{i2}$$,

$$\begin{aligned} \begin{aligned} E[(M_{i1}- M_{i2})^2|y_{i1},y_{i2}]&= y_{i1}(1-y_{i1})+y_{i2}(1-y_{i2})+(y_{i1}-y_{i2})^2 \\&= y_{i1}+y_{i2}-2y_{i1}y_{i2}. \end{aligned} \end{aligned}$$

Therefore, under A1, A2 and A3, the expectation of $z_i$ is given by

$$\begin{aligned} E(z_i ) = \frac{1}{K(K-1)} \left[ \text{ Var }(Y_{i1})+\text{ Var }(Y_{i2})+(E Y_{i1}- E Y_{i2})^2-K(\Lambda _{i1}+\Lambda _{i2}-2\Lambda _{i1}\Lambda _{i2}) \right] . \end{aligned}$$

Under the binomial assumption, $\text{ Var }(Y_{it})= K\Lambda _{it}(1-\Lambda _{it})$, and it follows that

17 $$\begin{aligned} \begin{aligned} E(z_i)&=\frac{1}{K(K-1)} \left[ K\Lambda _{i1}(1-\Lambda _{i1})+K\Lambda _{i2}(1-\Lambda _{i2})+K^2(\Lambda _{i1}-\Lambda _{i2})^2 \right. \\&\quad \left. -\,K[\Lambda _{i1}(1-\Lambda _{i1})+\Lambda _{i2}(1-\Lambda _{i2}) + (\Lambda _{i1}-\Lambda _{i2})^2]\right] \\&= (\Lambda _{i1}-\Lambda _{i2})^2. \end{aligned} \end{aligned}$$

Hence, the expected value of $z_i$ is zero under the null hypothesis of binomial dispersion as long as $x_{i1}'\beta = x_{i2}'\beta$.

One possible alternative to the null of a binomial variance is given by the beta-binomial model, where the variance is

18 $$\begin{aligned} \mathrm{\text{ Var }}(Y_i)= K \Lambda _{it}(1-\Lambda _{it})\left( 1+\eta \right) \end{aligned}$$

and $\eta$ is equal to $\eta =\frac{K-1}{\phi +1}> 0$. In this case, overdispersion originates from random unobserved heterogeneity.

### Case I: discrete covariates

Define the set of individuals with the same expectations over time, $A=\{i:\Lambda _{i1}=\Lambda _{i2}\}$, for which $E(z_i|i \in A)= 0$ holds. With time-invariant fixed effect $\alpha _i$ and a single regressor, the set *A* is equal to $\{i: x_{i1}=x_{i2}\}$. In general, the set *A* is broader, including all cases where $x_{i1}'\beta = x_{i2}'\beta$. In most cases, it will be possible to find such a set *A* if all covariates are finite discrete variables, assuming that the *x*-values are drawn from a stationary distribution. The test term for discrete $x_{it}$ is defined as:

19 $$\begin{aligned} \tau _A = {\hat{E}}(z_i|i \in A)= \frac{\sum _{i \in A} z_i}{ |A|}, \end{aligned}$$

where $\left| A\right|$ represents the number of elements in *A*. Under $H_0$, $\tau _A \xrightarrow {p } 0$. Further, by the central limit theorem (CLT), the statistic $\tau _A$ converges to a normal distribution,

20 $$\begin{aligned} \sqrt{\left| A\right| }( \tau _A -0) \xrightarrow {d } N(0, \sigma ^2_A), \end{aligned}$$

where $\sigma ^2_A= \text{ Var }(z_i| i \in A)$. In practice, $\sigma ^2_A$ is replaced by the sample variance ${\hat{\sigma }}^2_A$. So we reject the binomial distribution assumption at the $\alpha$% significance level if $\left| \frac{\tau _A}{{\hat{\sigma }}_A / \sqrt{\left| A\right| }}\right| \ge c_{1-\frac{\alpha }{2}}$, where the critical value *c* is the $1-\alpha /2$-percentile of the standard normal distribution.

Individuals in the set *A* do not contribute to the estimation of the Blogit CML model, since $x_{it}$ are cancelled out as fixed effects. Nonetheless, they are needed for generating our dispersion test. This nonparametric method to build a test is similar to finding proper cell estimators in matching theory, but likewise faces the curse of dimensionality. It is hard to find the set *A* when the dimension of $x_{it}$ becomes larger. If $\left| A\right|$ shrinks, the convergence rate $\sqrt{\left| A\right| }$ will decrease and the estimator $\tau _A$ will converge more slowly.

### Case II: continuous covariates

The set $A=\{i:\Lambda _{i1}=\Lambda _{i2}\}$ is empty or very small when $x_{i1}$ and $x_{i2}$ are continuous. A more general method uses a kernel estimator for the conditional expectation $E(z_i| \Lambda _{i1}-\Lambda _{i2}=0)$. The main idea is to put more weight on individuals with smaller $\left| \Lambda _{i1}-\Lambda _{i2}\right|$. Since we do not observe the underlying expectations $\Lambda _{it}$ directly, we find the set *A* by using observables $x_{it}$. Under the assumption of a single scalar regressor and time-invariant unobserved heterogeneity, we can decompose the conditional expectation (17) by a Taylor expansion at $x_{i2}$,

$$\begin{aligned} \begin{aligned} (\Lambda _{i1}-\Lambda _{i2})^2&= [ \Lambda (x_{i1}\beta +\alpha _i)-\Lambda (x_{i2}\beta +\alpha _i)]^2\\&= [\Lambda '(x_{i2}\beta +\alpha _i)\beta \left( x_{i1}-x_{i2} \right) \\&\quad + \frac{\Lambda ''(x_{i2}\beta +\alpha _i)}{2!}\beta ^2 \left( x_{i1}-x_{i2}\right) ^2 +o(\left( x_{i1}-x_{i2}\right) ^2)]^2 \\&= [\Lambda '(x_{i2}\beta +\alpha _i)\beta \left( x_{i1}-x_{i2} \right) ]^2 + o(\beta ^2( x_{i1}-x_{i2})^2), \end{aligned} \end{aligned}$$

Denote $$\Delta _i= (x_{i1}-x_{i2}) \beta $$,

$$\begin{aligned}E(z_i| \Lambda _{i1}-\Lambda _{i2}) =( \Lambda _{i1}-\Lambda _{i2})^2 = ( \Lambda _{i2}' \Delta _i)^2 + o(\Delta _i^2). \end{aligned}$$

As the fixed effect $\alpha _i$ is cancelled out, an alternative conditional expectation function is given by $\Delta _i$,

$$\begin{aligned} \tau (\Delta )= E(z_i| \Delta _i=\Delta , X_i)= (\Lambda _{i2}' \Delta )^2. \end{aligned}$$

Then, under the binomial assumption,

$$\begin{aligned} E(z_i| \Lambda _{i1}-\Lambda _{i2}=0) = \tau (0)=0. \end{aligned}$$

The result generalizes to a vector-valued *x*, in which case $\Delta _i= (x_{i1}-x_{i2})' \beta$.

The next step is to build a kernel estimator for $\tau (0)$. One conditional moment estimator for $\tau (\Delta )$ is $\hat{\tau }(\Delta )= \frac{\sum _{i=1}^N K(\frac{\Delta _i- \Delta }{h}) z_i}{\sum _{i=1}^N K(\frac{\Delta _i- \Delta }{h}) }$, where *h* is the kernel bandwidth for $\Delta _i$ and $K(\frac{\Delta _i- \Delta }{h})$ is the kernel function. For a given sample, $\Delta _i$ needs to be replaced by ${\hat{\Delta }}_i= (x_{i1}-x_{i2})\hat{\beta }$, where $\hat{\beta }$ is estimated. We can use the Blogit CML estimator for estimation, as it is consistent under the binomial null hypothesis. We construct a local estimate ${\hat{\tau }}$ for the object of interest $\tau (0)$ (see Pagan and Ullah 1999):

$$\begin{aligned} \begin{aligned} {\hat{\tau }}&= \frac{\sum _{i=1}^N K(\frac{{\hat{\Delta }}_i}{h}) z_i}{\sum _{i=1}^N K(\frac{{\hat{\Delta }}_i}{h}) } = \sum _{i=1}^N w_{ni} z_{i}, \quad w_{ni}=\frac{ K(\frac{{\hat{\Delta }}_i}{h}) }{\sum _{i=1}^N K(\frac{{\hat{\Delta }}_i}{h}) }, \end{aligned} \end{aligned}$$

The Gaussian function $K(\frac{{\hat{\Delta }}_{i}}{h})= \frac{1}{\sqrt{2\pi }} \exp (-\frac{({\hat{\Delta }}_{i}/h)^2}{2})$ is chosen for simplicity.

### Asymptotic properties

Let $f=f(\Delta =0)$ denote the continuous density function of the random variable $\Delta$ at point 0. The kernel density estimator ${\hat{f}}$ for f is

$$\begin{aligned} {\hat{f}}= \sum _{i=1}^N \frac{K(\frac{{\hat{\Delta }}_i}{h})}{nh}. \end{aligned}$$

In addition, rewrite $z_i$ as the sum of its conditional expectation $E(z_i| \Delta _i)=\tau (\Delta _i)$ and an error term $u_i$, such that

$$\begin{aligned} z_i= \tau (\Delta _i)+u_i=(\Lambda '_{i2} \Delta _i)^2+u_i \end{aligned}$$

where $$E(u_i|\Delta _i, X_i)=0$$ and $$\text{ Var }(u_i|\Delta _i, X_i)= \sigma ^2 $$.

The estimator ${\hat{\tau }}$ is a combination of ${\hat{f}}$ and $z_i$

$$\begin{aligned} {\hat{\tau }} = \frac{\sum _{i=1}^N \frac{1}{nh} K\left( \frac{\hat{\Delta }_i}{h}\right) z_i}{\sum _{i=1}^N \frac{1}{nh} K\left( \frac{\hat{\Delta }_i}{h}\right) }= \frac{1}{{\hat{f}}} \sum _{i=1}^N \frac{1}{nh} K\left( \frac{{\hat{\Delta }}_i}{h}\right) z_i =\frac{1}{{\hat{f}}} \sum _{i=1}^N \frac{1}{nh} K\left( \frac{{\hat{\Delta }}_i}{h}\right) (\Lambda '_{i2} \Delta _i)^2+u_i. \end{aligned}$$

The expectation of ${\hat{\tau }}$ is

$$\begin{aligned} \begin{aligned} E({\hat{\tau }})&= E\left( \frac{1}{{\hat{f}}} \sum _{i=1}^N \frac{1}{nh}K\left( \frac{{\hat{\Delta }}_i}{h}\right) \left( \Lambda '_{i2} \Delta _i\right) ^2 + \frac{1}{{\hat{f}}} \sum _{i=1}^N \frac{1}{nh}K\left( \frac{{\hat{\Delta }}_i}{h}\right) u_i\right) \\&= \int \int \frac{1}{h {\hat{f}}} K(\nu ) (\Lambda '_{i2})^2 (h\nu )^2 f(h\nu , \Lambda _{i2}) h \mathrm{d}\nu \mathrm{d}\Lambda _{i2}\\&\quad + {\hat{E}}(u_i| \hat{\Delta }=0), \quad \text {where we replace } \Delta = h\nu \\&= h^2 \int \int K(\nu )(\nu )^2 (\Lambda '_{i2})^2 \frac{f(\nu , \Lambda _{i2}) }{{\hat{f}}} \mathrm{d}\nu \mathrm{d}\Lambda _{i2}\\&= h^2 \mu _2 E[(\Lambda '_{i2})^2 | \Delta =0], \quad \text {where } \mu _2= \int K(\nu ) (\nu )^2 \mathrm{d}\nu \end{aligned} \end{aligned}$$

We therefore obtain a bias

21 $$\begin{aligned} \text {Bias}({\hat{\tau }}) =E({\hat{\tau }}) - \tau (0)= E({\hat{\tau }})= h^2 \mu _2 E[(\Lambda '_{i2})^2 | \Delta =0 ], \end{aligned}$$

that is proportional to $h^2$.

To guarantee consistency of the estimator ${\hat{\tau }}_n$, convergence of the mean square error to zero is required. The MSE is equal to $\text {MSE}({\hat{\tau }}) =\text {Bias}({\hat{\tau }})^2+ \text{ Var }({\hat{\tau }})$. So the bias for $\tau _n$ should decrease to zero, as *n* increases:

22 $$\begin{aligned} h^2 \longrightarrow 0, \quad \text {as } n \longrightarrow \infty . \end{aligned}$$

Besides the convergence condition for bias, we also consider the asymptotic performance of the variance of ${\hat{\tau }}$. Using a result on the variance of conditional expectations from Pagan and Ullah (1999), we obtain:

23 $$\begin{aligned} \text{ Var }({\hat{\tau }})= \frac{\sigma ^2}{nhf} \int K^2(\nu )\mathrm{d}\nu , \quad \text{ Var }({\hat{\tau }}) \propto \frac{1}{nh}. \quad \text {If } n \longrightarrow \infty , \quad \frac{1}{nh} \longrightarrow 0. \end{aligned}$$

To make sure that the MSE converges at the fastest speed, $\text {bias}^2$ and variance should converge at the same rate: $h^4 \propto \frac{1}{nh}$. Otherwise, the slower speed dominates the convergence rate. Thus, *h* is of order $h \propto n^{-\frac{1}{5}}$ and by the central limit theorem,

24 $$\begin{aligned} \sqrt{nh} ({\hat{\tau }}- E({\hat{\tau }})) \xrightarrow {d } N(0, f^{-1}\sigma ^2 \int K^2(\nu )\mathrm{d}\nu ) \end{aligned}$$

Here, $\sigma ^2= \text{ Var }(z_i^2|\Delta =0)$, with the same definition as in the discrete case (Eq. 20). In practice, we standardize $\Delta _{i}$ at first and set bandwidth $h'=0.9n^{-\frac{1}{5}}$. The approximate bias is calculated by ${\hat{E}}({\hat{\tau }})= \sum _{i=1}^N w_{ni} (y_{i2}(1-y_{i2})\hat{\Delta })^2$, $\sigma ^2$ is replaced by ${\hat{\sigma }}^2= \sum w_{ni} (z_i- \tau ({\hat{\Delta }}_i))^2$ and ${\widehat{\text{ Var }}}({\hat{\tau }})= \frac{\sigma ^2}{{\hat{f}}^2 }\frac{\sum _{i=1}^N K^2 (\nu )}{n^2 h^2}$. Hence, $\frac{{\hat{\tau }} - {\hat{E}}(\hat{\tau })}{\sqrt{{\widehat{\text{ Var }}}({\hat{\tau }})}}$ can be used as a *t*-test.

### Multiple periods

The test can be extended to multiple time periods. With $T=2$, there is a single moment condition for $E(z_i|\Delta _i=0)$ that can be tested. For $T>2$, one possibility is to combine $T-1$ such moment conditions into a single test statistic.

In the discrete case, for set $A_t=\{i: x_{i,t}=x_{i,t+1} \}$,

$$\begin{aligned} g_{i,t}= \frac{(Y_{i,t}-Y_{i,t+1})^2-K(M_{i,t}-M_{i,t+1})^2}{K(K-1)}, \quad t= 1 ,\ldots , T-1. \end{aligned}$$

$g_{i,t}$ is empty if $x_{i,t} \ne x_{i,t+1}$. As we derived before, $E(g_{i,t})=0$.

In matrix form,

$$\begin{aligned}g_i= \begin{pmatrix} g_{i,1}\\ \ldots \\ g_{i,T-1} \end{pmatrix}, \quad \text {and the sample mean is }{\bar{g}}_n= \begin{pmatrix} \frac{1}{n_1} \sum \limits _{i=1}^{n_1} g_{i,1}\\ \ldots \\ \frac{1}{n_{T-1}} \sum \limits _{i=1}^{n_{T-1}} g_{i,T-1} \end{pmatrix}, \end{aligned}$$

with $n_t= |A_t|$, the cardinality of set $A_t$. Denote $n= (n_1, \ldots , n_{T-1})'.$ To calculate the sample variance-covariance matrix ${\hat{S}}$, we replace off-diagonal elements with pairwise sample covariances and diagonal ones with $g_t$ sample variances. A test statistics can be derived

$$\begin{aligned} J= (\sqrt{n} \circ {\bar{g}}_n)' {\hat{S}}^{-1} (\sqrt{n} \circ {\bar{g}}_n). \end{aligned}$$

In the continuous case, moment conditions are

$$\begin{aligned} g_{i,t}= \frac{ K\left( \frac{{\hat{\Delta }}_{i,t}}{h}\right) (z_{i,t}-\tau (\hat{\Delta }_{i,t}))}{\sum _{i=1}^N \frac{1}{n} K\left( \frac{\hat{\Delta }_{i,t}}{h}\right) }, \quad t= 1 ,\ldots , T-1 \end{aligned}$$

where $$ {\hat{\Delta }}_{it}=(x_{it}-x_{i,t+1}){\hat{\beta }}$$ and $$\tau ({\hat{\Delta }}_{i,t})= (y_{i,t+1}(1-y_{i,t+1}) {\hat{\Delta }}_{it})^2$$. Under the null hypothesis, $$E(g_{i,t})=0. $$

These moment conditions can be written in matrix form for individual $i =1,\ldots ,N$ as:

$$\begin{aligned}g_i= \begin{pmatrix} g_{i,1}\\ \ldots \\ g_{i,T-1} \end{pmatrix}, \quad \text {and the sample mean is }{\bar{g}}_N= \begin{pmatrix} \frac{1}{N} \sum \limits _{i=1}^N g_{i,1}\\ \ldots \\ \frac{1}{N} \sum \limits _{i=1}^N g_{i,T-1} \end{pmatrix}. \end{aligned}$$

Let ${\hat{S}}$ denote the sample variance:

$$\begin{aligned} {\hat{S}}= \frac{1}{N} \sum \limits _{i=1}^N g_i g_i'- {\bar{g}}_n \bar{g}_n', \end{aligned}$$

Since ${\bar{g}}_N \xrightarrow {p } E(g_i) =0,$ a test statistic is given by

$$\begin{aligned} J= N \,{\bar{g}}_N' {\hat{S}}^{-1} {\bar{g}}_N = (\sqrt{N} \cdot \bar{g}_N)' {\hat{S}}^{-1} (\sqrt{N} \cdot {\bar{g}}_N) \end{aligned}$$

Therefore, $$\sqrt{N} \cdot {\bar{g}}_N \xrightarrow {d } N(0,S) $$, $${\hat{S}} \xrightarrow {p } S$$, and $$J \xrightarrow {d } \chi ^2_{T-1}$$.

The Chi-square test rejects the binomial distribution assumption at the $\alpha$% significance level if $J \ge \chi ^2_{\alpha }(T-1)$.

### Simulation study

We conduct a number of simulation experiments to examine the performance of these tests under two scenarios. In the first setting, explanatory variables are discrete (in fact, there is a single binary regressor, to keep things as simple as possible), while the explanatory variable is continuous in the second. The remaining aspects of the DGP regarding fixed effects, expectation functions and parameters setting are the same as those in Sect. 2.3.

**Table 2:** Simulation results for rejection rates when *x* is discrete

$N=100$	Overdispersion (%)	$T=2$	$T=5$	$T=10$	$N=500$	Overdispersion (%)	$T=2$	$T=5$	$T=10$
$K=2$	0	0.062	0.062	0.057	$K=2$	0	0.032	0.062	0.055
	10	0.080	0.093	0.130		10	0.167	0.258	0.358
	50	0.502	0.817	0.927		50	0.992	1	1
$K=5$	0	0.056	0.064	0.053	$K=5$	0	0.059	0.058	0.066
	10	0.044	0.068	0.066		10	0.102	0.147	0.187
	50	0.225	0.449	0.579		50	0.885	0.999	1
	100	0.603	0.929	0.990		100	0.999	1	1
	200	0.938	0.999	1		200	1	1	1
$K=10$	0	0.052	0.053	0.069	$K=10$	0	0.053	0.055	0.055
	10	0.052	0.060	0.071		10	0.075	0.118	0.185
	50	0.204	0.395	0.497		50	0.836	0.993	1
	100	0.492	0.878	0.972		100	1	1	1
	200	0.869	1	1		200	1	1	1

$x_{it}$ is a binary variable with 50% probability equal to 0 or 1. The remaining DGP is the same as in Table 1; the null hypothesis is that of binomial dispersion

Table 2 presents rejection rates, i.e. the relative number of times that our test rejects the binomial assumption over 1000 replications, when *x* is discrete. $x_{it}$ is either 0 or 1 with equal probability. In this case, $\Pr (x_{i1}= x_{i2})= 50\%$, and on average half of the observations will be in the set *A* of individuals with the same expectations over time and thus informative for computing the test statistic. As before, the number of time periods increases from $T=2$ to $T=10$, and binomial parameter from $K=2$ to $K=10$.

The first row of each subpanel shows results without overdispersion, i.e. sampling from a binomial DGP applies. In this case, the rejection rates are equivalent to the proportion of type-I errors and ideally should be close to the nominal size of the test, in this case 5%. The lower part of each subpanel shows the rejection rates under $H_0$ when $H_0$ is false, i.e. the power.

When we implemented the multi-period discrete test as described in Sect. 3.4, we found that the size of the test was seriously distorted when *T* was large. For $T=10$, the rejection rates under the binomial assumption were 44.7% for $N=100$, and 12.9% for $N=500$. For larger *N*, there is a convergence to the nominal size, but it is rather slow (e.g. 8.5% rejection rate for $N=1000$). The reason for this test behaviour is the poor estimation of the covariance elements of the weighing matrix ${\hat{S}}$. For example, when $T=3$, the covariance between $g_{i,1}$ and $g_{i,2}$ is estimated based on the small subset of observations for which $x_{i1}=x_{i2}=x_{i3}$. The imprecise estimation of the covariances for small *N* leads to a large sampling variability of ${\hat{S}}$, and this problem increases with *T*. As an alternative, we therefore show in Table 2 simulation results, where all off-diagonal elements of ${\hat{S}}$ were set to zero, leading to a better performance of the test in small samples. We also note that the continuous, kernel-based test does not suffer from this problem.

Reassuringly, we find that the test has some power against the alternative of rather modest overdispersion (10%), in particular for $N=500$, $K=2$ and $T=10$, where around 36% of wrong null hypotheses are rejected. As the dispersion degree increases, the power of the test also grows, and it reaches 100% for DGPs where overdispersion, the number of observations, and the number of time periods are large.

**Table 3:** Simulation results for rejection rates when *x* is continuous

$N=100$	Dispersion (%)	$T=2$	$T=5$	$T=10$	$N=500$	Dispersion (%)	$T=2$	$T=5$	$T=10$
$K=2$	0	0.053	0.063	0.097	$K=2$	0	0.062	0.047	0.056
	10	0.070	0.095	0.185		10	0.138	0.223	0.394
	50	0.424	0.684	0.899		50	0.948	1	1
$K=5$	0	0.05	0.053	0.078	$K=5$	0	0.042	0.05	0.057
	10	0.044	0.063	0.110		10	0.083	0.105	0.190
	50	0.239	0.512	0.805		50	0.772	0.992	1
	100	0.608	0.958	0.999		100	0.996	1	1
	200	0.927	0.991	0.995		200	1	1	1
$K=10$	0	0.041	0.062	0.091	$K=10$	0	0.052	0.056	0.043
	10	0.041	0.057	0.086		10	0.073	0.094	0.179
	50	0.190	0.402	0.673		50	0.677	0.988	1
	100	0.482	0.908	0.997		100	0.991	1	1
	200	0.873	0.999	0.999		200	1	1	1

Results for 1000 Monte Carlo replications; standard deviations in parentheses. In each period, the number of observation is 100. $x_{it} \sim U[-1,1]$ . $\beta _1= 2$ and $\alpha _i = \sqrt{T} {\bar{x}}_i + N(0,1)$. Overdispersion factor $\frac{k-1}{\phi +1 }$ represents dispersion degree of variance. Overdispersion degree 0% is generated by binomial distribution, and positive dispersion degree is generated by a beta-binomial DGP

In Table 3, we show the results for the kernel weighted test statistics for continuous regressors. $x_{it}$ is drawn from a uniform distribution with positive support between -1 and 1, with mean 0 and variance 1/3. The general patterns regarding type-I errors and power of the tests are mostly similar to those of Table 2. As in Table 2, the power of the test tends to decrease in *K*, for a given overall degree of overdispersion, but this tendency is more uniform in the continuous version of the test. This indicates that the power of the test reacts differently to the two parameters driving overdispersion, and in particular that it is more sensitive to increases in $\phi$ rather than *K*. The combined results from our simulation experiments are reassuring: on the one hand, modest amounts of overdispersion cause only minor bias of the Blogit CML estimator; on the other hand, the test we derive has good power properties against medium- or high-dispersion alternatives to the binomial assumption.

## Application to labour supply

In this illustrative application of the binomial estimator and overdispersion test, we re-consider the association between fertility and female labour supply. Data are from the Swiss Household Panel (SHP) for the years 2012–2016. The SHP is an ongoing longitudinal survey of people residing in Switzerland. It collects information on a large range of topics on living conditions, both objective and subjective, including work, fertility and health. We restrict the analysis to women aged 25–45, who participated in the survey at least twice during the 5-year period. This gives us a panel of 5854 person-year observations for 1712 different women.

There exists a huge literature modelling female labour supply, a large part of which is devoted to the endogeneity of the fertility decision. We want to make here a different point, namely that the labour supply outcome, i.e. the amount of time a women decides to spend in market work, fits in principle into the empirical framework discussed in this paper and hence can be analysed using the methods proposed in this paper: empirically, the amount of days or hours worked is discrete, and it has a lower bound of zero, as well as an upper bound, and can thus be expressed as a proportion.

Modelling labour supply as a fraction of time may be promising in particular in institutional settings, where employment contracts offer various part-time options. A case in point is Switzerland, where vacancies are advertised, and work contracts are written using full-time fractions. For instance, 60% work-time means that the worker is employed for the equivalent of 3 days per week and also is paid 60% of a full-time salary. In practice, the large majority of agreed-upon work-time percentages are multiples of 10%.

**Fig. 3:**
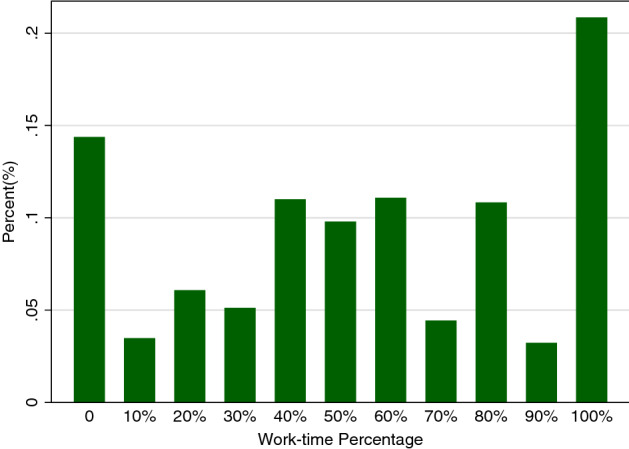
Distribution of Work-time percentage in 2016 *Source:* Swiss Household Panel 2012–2016. Women aged 25–45. “Work-time percentage” denotes the contracted work-time. For example, $60\% = 3$ full days per week; 0 indicates non-work, 100% full-time work

Figure 3 shows the distribution of work-time percentages for the sample of women extracted from the Swiss Household Panel. Here, the data are pooled over the five years. The relative frequency of zeros is 14.4%, meaning that the estimated participation rate in our sample for this age group is 85.6%, a number very close to the official statistic published by the Federal Statistical Office (BfS 2016). Although there are peaks for non-work and for full-time work, all intermediate values are present in the data.

**Fig. 4:**
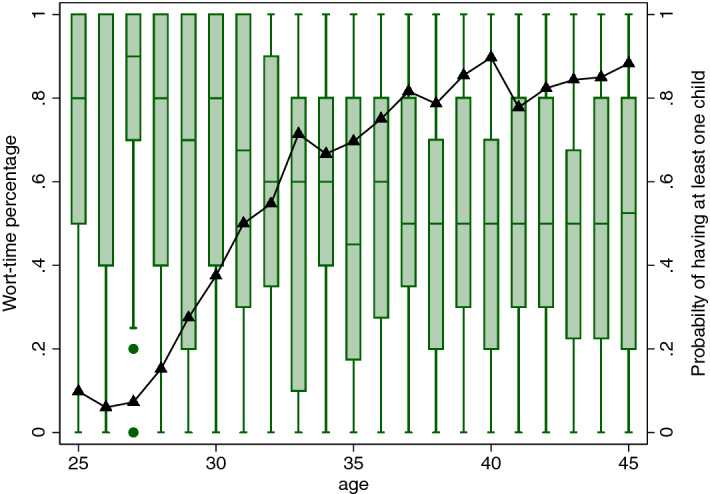
Work-time percentage and motherhood by age in 2016. *Notes:* The green bar is the box plot of the work-time percentage by age in 2016; the black triangle denotes the probability of having at least one child in 2016. (Color figure online)

In particular, Fig. 3 documents that for Switzerland, the vast majority of women does not work full-time. A question one can then ask is: How does the work-time percentage vary with the presence of children in the household? Box-plots in Fig. 4 show, for our data, a clear negative association between work and children. The median work-time percentage drops from 80% or higher for those aged 30 or below to 50% for women in their early 40s. At the same time, older women are more likely to have children.

The key assumption of the following analysis is that we can treat 10-times the work-time percentage as a binomial variable with outcomes $0,1,\ldots ,10$, where the mean will be modelled as a function of covariates as well as individual-specific time-invariant fixed effects.

It is of course difficult to imagine the work-time decision as literally arising from an underlying sequence of *K* independent Bernoulli trials. Nevertheless, the binomial model can provide a useful approximation to the distribution of work-time percentages, in particular since it conditions on fixed effects and hence is compatible with an observed unconditional “W-shaped” outcome distribution as observed in Fig. 3. Our illustrative application abstracts from additional complexities, such as potential differences between the participation decision (i.e. the extensive margin) and the intensive margin, and, beyond the inclusion of individual fixed effects, the endogeneity of the fertility decision. Clearly, these are important concerns, and they should be addressed in further extensions of the approach.

### Results

Table 4 provides some descriptive statistics (means and standard deviations) for both the dependent and the explanatory variables used in the estimation. The average work-time percentage is 56%, with a standard deviation of 0.34. Under the binomial assumption, the standard deviation for a fraction with a mean of 0.56 is equal to $\sqrt{0.56 (1-0.56)/10} = 0.157$, substantially below the observed standard deviation of 0.346. Hence, there is evidence of overdispersion at the marginal level.

**Table 4:** Descriptive statistics ($NT= 5854$) *Source*: Swiss Household Panel 2012–2016, own calculations

	Mean	SD
Work-time percentage	0.557	0.346
Age	36.30	6.01
Children ($\hbox {yes}=1$)	0.631	0.482
Partner ($\hbox {yes}=1$)	0.584	0.492
Self-rated health	3.114	0.610
(0: worst to 4: best)		
French speaking ($\hbox {yes}=1$)	0.293	0.455
Italian speaking ($\hbox {yes}=1$)	0.043	0.204

Women have an average age of 36.3 years, and 63.1% report having at least one child in the year they are surveyed. For 58.4% of person-year observations, there is a partner present in the household. The health status is captured by a 5-point scale for self-assessed health, where 0 means “not well at all” and 4 means “very well”. We treat it as a cardinal scale for simplicity, and also abstract from its potential endogeneity to working or having children. Finally, we include information on language region. There is quite a bit of evidence that work-norms differ between the French and the German-speaking parts of Switzerland, with some stigma attached to working mothers, in particular during the first years of the child’s life (see Steinhauer 2018). This stigma seems to be stronger in the German-speaking part of Switzerland (65% or our sample) but less so in the French-speaking part (29% of our sample).

Our final estimation model includes four year dummies, age-squared (the linear age term is dropped; alternatively, one could identify the linear age effect by setting a second year effect equal to zero), indicators for the presence of a child and partner, and the health variable. Since language region is mostly constant over time, it is near-collinear with the fixed effects when applying the Blogit CML or Blogit DV estimators, and we therefore only include its interaction with the child-indicator variable.

As is the case for the binary logit model with fixed effects, DV estimation of the binomial model is subject to the perfect prediction problem (see, for example, Kunz et al. 2018). Outcomes for women, whose work-time percentage is either zero or one in each year, are perfectly predicted, meaning that the associated dummy coefficient will tend to minus or plus infinity, respectively. For the Blogit CML, perfectly prediction formally does not arise as the $\alpha _i$’s are not estimated. However, all such observations have mechanically a log-conditional likelihood value of zero and thus do not contribute to estimation of $\beta$ either. To use the same estimation sample everywhere, we right away drop all perfectly predicted outcomes, leading to a final sample size of 4661 person-year observations for the work-time percentage model.

**Table 5:** Determinants of female labour supply (2012–2016) *Source*: Swiss Household Panel

	Work-time percentage		Work (yes/no)	
	Blogit CML	Blogit DV	Logit CML	Logit DV
Age-squared	$-$ 0.001	$-$ 0.001	0.005	0.007
	(0.002)	(0.002)	(0.005)	(0.008)
Self$-$ rated health	0.071	0.073	0.448	0.642
	(0.038)	(0.039)	(0.153)	(0.220)
Partner (yes=1)	0.322	0.333	1.371	1.927
	(0.253)	(0.260)	(0.658)	(0.952)
Children (yes=1)	$-$ 2.097	$-$ 2.160	$-$ 1.975	$-$ 2.771
	(0.276)	(0.286)	(0.956)	(1.381)
Children $\times$ Partner	$-$ 0.824	$-$ 0.848	$-$ 2.216	$-$ 3.188
	(0.260)	(0.268)	(1.056)	(1.510)
Children $\times$ French	1.152	1.194	1.876	2.777
	(0.405)	(0.418)	(1.089)	(1.619)
Children $\times$ Italian	$-$ 0.247	$-$ 0.266		
	(0.720)	(0.754)		
Year 2013	0.143	0.147	$-$ 0.018	$-$ 0.018
	(0.147)	(0.151)	(0.465)	(0.647)
Year 2014	0.246	0.253	$-$ 0.147	$-$ 0.265
	(0.278)	(0.285)	(0.820)	(1.143)
Year 2015	0.338	0.347	$-$ 0.247	$-$ 0.408
	(0.412)	(0.423)	(1.205)	(1.677)
Year 2016	0.387	0.397	$-$ 0.449	$-$ 0.717
	(0.545)	(0.560)	(1.577)	(2.204)
Number of person-years	4661	4661	1071	1071
Number of persons	1334	1334	295	295
Log pseudo-likelihood	$-$ 23,183.6	$-$ 1838.3	$-$ 358.8	$-$ 595.9
Fixed effects	Yes	Yes	Yes	Yes

*Blogit CML* denotes the binomial logit conditional maximum likelihood estimator; *Blogit DV* is the binomial logit estimator with dummy variables for each individual; *Logit CML* and *Logit DV* are the corresponding estimators for the binary logit model

Regression results are given in Table 5. The first column shows the estimated coefficients from the Blogit CML and the second those from the Blogit DV model. The last two columns add corresponding (binary) logit models for the extensive margin model (work yes/no), again using alternatively the CML or DV estimators. Standard errors are clustered at the individual level. The linear age term has been dropped due to collinearity in a model with individual and year fixed effects.

When interpreting magnitudes, we note the recent suggestion by Kemp and Santos Silva (2016) and focus on expected (semi-) elasticities. These can be estimated without knowledge of $\alpha _i$ and are thus very suitable for our conditional maximum likelihood approach. For the binomial proportion model with $E(y_{it}|x_{it},\alpha _i) = \Lambda _{it}$, we obtain

$$\begin{aligned} \partial \log E(y_{it}|x_{it},\alpha _i)/\partial x_{it} = \beta (1- \Lambda _{it}) \end{aligned}$$

A good estimator of the overall mean of $\Lambda _{it}$ is the sample mean of the outcome, ${\bar{\Lambda }} = {\bar{y}} =0.55$, so that the CML estimators ${\hat{\beta }}$ can be multiplied by 0.45 to obtain an estimate of the population average semi-elasticities with respect to changes in the associated covariate.

From columns (1) and (2) of Table 5, we find a large negative association between having a child and the amount of work. The point estimate of the main effect is about -2, which means that not having a child increases the expected work-time percentage by about 90 percent. This effect is highly statistically significant, as are two of the three interaction effects: having a child reduces the work-time percentage more if a partner is present than otherwise, underlining the relevance of pecuniary motives for work, and the need to “make ends meet”. The labour supply response of women to having children is about half as large for French-speaking women as it is for German speakers, corroborating the social norm results found in the earlier literature (Steinhauer 2018).

In this application, the Blogit CML and the Blogit DV results are very similar. The DV results are always a bit larger in absolute value, but the difference never exceeds 5%. This resonates with our simulation results, because both *T* and *K* are relatively large. Nevertheless, the joint test for the binomial assumption derived in Sect. 3.3 indicates a clear rejection (test value of 37.7 with a $\chi ^2_{0.95}$ critical value of 9.5). This rejection result due to overdispersion was already foreshadowed, although not logically implied because of the conditional nature of the test, by the high proportion of no work (zero) and full-time work (100%) as evident in Fig. 1. However, we know from the simulation results (Tables 1, 2) that even with 50% overdispersion, the bias of the Blogit CML is small for $K=10$ and $T=5$, a setting similar to the current application. At the same time, the probability of rejecting the wrong $H_0$ is very close to 1 (see Table 3). On a practical note, the CML estimator can be computed much faster than the DV estimator, by a factor of about 10 in our case. The speed problem of DV models would be exacerbated in applications with more cross-sectional units, to the point where computation of the Blogit DV estimator may become infeasible in the current Stata/R setting.

In the last two columns of Table 5, we allow for a comparison with results from a more conventional binary logit extensive margin estimator. A first point to note is that the effective sample becomes much smaller, since all observations with variation in the positive range only, i.e. percentages between 10 and 100%, are now coded as “1” and thus become perfectly predicted. Their variation does not contribute to estimation, the usable sample size drops by 3/4, and the standard errors of the estimated coefficients increase accordingly. We had to drop the interaction between speaking Italian and having children, as it could not be estimated in the reduced sample.

The estimated coefficients tend to be substantially larger, but they are not directly comparable. To obtain the implied expected semi-elasticities for the probability of work, coefficients need to be multiplied by the non-participation rate, 0.145 in this case, compared to a factor of 0.45 applicable in the first two columns. Based on the CML estimates, some of the extensive margin semi-elasticities are smaller than the overall semi-elasticities (like the main effect of having a child), and some of them larger (such as self-rated health). In terms of statistical significance, we find that the health and partner coefficients were not significant in the work-time percentage model, but they are in the participation model. And in terms of point estimates, the interaction between speaking French and having children just offsets the main effect of having at least one child, meaning that there is no difference in participation probabilities for French-speaking mothers and non-mothers, although some labour supply responsiveness was found in the work-time percentage model for the combined extensive and intensive margin effect. Also, the participation model suffers from a massive incidental parameters bias, since the point estimates for the DV estimator exceed those of the CML estimator by 50% on average.

## Concluding remarks

Although Machado (2004) introduced the fixed effects binomial model as a method for proportions of successes in a sequence of Bernoulli trials, it can be used for discrete bounded outcomes, or fractions, more generally. However, it remained an open question whether or not the conditional binomial logit maximum likelihood estimator is robust to misspecification. In this paper, we focus on the consequences of overdispersion as it originates, for instance, from neglected unobserved heterogeneity. We show in simulation experiments that the Blogit CML estimator maintains a rather good performance even if the binomial model is misspecified, as long as the length of the panel *T* is sufficiently large, or the degree of overdispersion is modest.

We then derive a test of the null hypothesis that the binomial assumption is valid, based on departures from the implied binomial variance function. The test computes the variance of within-individual outcome differences. For the subset of observations whose regressors do not change over time, the mean difference is zero (and close to zero if regressors do not differ “too much”) and it is possible to construct and compare two variance estimates, one with and one without the binomial assumption, that both do not depend on fixed effects. This is essential, as fixed effects are not estimated by the Blogit CML estimator. Our simulation experiments show that the test has good power properties against the alternative of medium or large degrees of overdispersion. But these are exactly the cases where the bias of the Blogit CML estimator becomes noticeable.

We study in our empirical application an outcome related to women’s work behaviour, namely the contracted work-time percentage. In our sample of mid-aged women obtained from the Swiss Household Panel, 65% of all women report working part-time, i.e. a percentage between 10 and 90%. The empirical analysis using the fixed effects binomial logit model predicts substantially different work-time percentages for mothers and non-mothers. Having a partner makes the difference more pronounced, whereas speaking French reduces it. We show how these coefficients can be interpreted in terms of expected semi-elasticities even if the fixed effects are not estimated. In comparison with the fixed effects logit estimation for the participation model, much fewer observations are lost in the work-time percentage model due to perfect prediction, contributing to a much more precise estimation of the model parameters.

In future work, we will consider alternative estimators that could be pursued if the binomial null hypothesis is rejected. If the logit conditional expectation function is to be kept, a binomial logit correlated random effects model is a possible approach. Such a model would explicitly account for overdispersion, by assuming for instance that random, time-varying unobserved heterogeneity follows a normal distribution with mean depending on the regressors.

## Appendix A

See Table 6.

**Table 6:** Simulation results $N=100$

$N=100$	Overdispersion (%)	$T=2$			$T=5$			$T=10$		
		Blogit CML	Blogit DV	Pooled logit	Blogit CML	Blogit DV	Pooled logit	Blogit CML	Blogit DV	Pooled logit
K=2	0	2.049	2.880	2.242	2.003	2.280	1.986	2.006	2.134	1.877
		(0.419)	(0.621)	(0.255)	(0.178)	(0.211)	(0.145)	(0.118)	(0.128)	(0.101)
	10	2.100	2.968	2.225	2.036	2.320	1.988	2.010	2.138	1.876
		(0.419)	(0.632)	(0.263)	(0.192)	(0.227)	(0.156)	(0.121)	(0.131)	(0.105)
	50	2.516	3.674	1.898	2.179	2.484	1.908	2.073	2.204	1.937
		(0.822)	(1.283)	(0.399)	(0.360)	(0.429)	(0.279)	(0.246)	(0.266)	(0.222)
$K=5$	0	2.012	2.279	2.243	2.000	2.101	1.990	2.002	2.051	1.872
		(0.233)	(0.272)	(0.190)	(0.111)	(0.118 )	(0.104)	(0.073)	(0.075)	(0.076)
	10	2.033	2.303	2.239	2.013	2.115	1.990	2.010	2.059	1.875
		(0.252)	(0.295)	(0.184)	(0.120)	(0.128)	(0.115)	(0.079)	(0.081)	(0.078)
	50	2.139	2.431	2.205	2.054	2.159	1.971	2.032	2.081	1.882
		(0.300)	(0.354)	(0.214)	(0.139)	(0.149)	(0.122)	(0.086)	(0.088)	(0.083)
	100	2.320	2.657	2.140	2.111	2.219	1.958	2.052	2.102	1.898
		(0.402)	(0.485)	(0.253)	(0.176)	(0.188)	(0.147)	(0.116)	(0.119)	(0.104)
	200	2.721	3.167	1.887	2.231	2.345	1.891	2.115	2.167	1.948
		(0.736)	(0.910)	(0.354)	(0.306)	(0.326)	(0.246)	(0.208)	(0.214)	(0.186)
$K=10$	0	2.005	2.129	2.241	2.001	2.050	1.989	2.000	2.025	1.871
		(0.157)	(0.169)	(0.153)	(0.078)	(0.081)	(0.091)	(0.052)	(0.052)	(0.063)
	10	2.017	2.142	2.237	2.004	2.053	1.986	2.002	2.026	1.868
		(0.174)	(0.187)	(0.158)	(0.082)	(0.084)	(0.091)	(0.055)	(0.055)	(0.063)
	50	2.065	2.195	2.241	2.026	2.076	1.984	2.012	2.036	1.869
		(0.202)	(0.219)	(0.175)	(0.098)	(0.101)	(0.100)	(0.064)	(0.065)	(0.072)
	100	2.139	2.275	2.214	2.051	2.102	1.979	2.025	2.049	1.876
		(0.239)	(0.260)	(0.183)	(0.109)	(0.113)	(0.106)	(0.074)	(0.075)	(0.076)
	200	2.293	2.443	2.146	2.105	2.157	1.963	2.049	2.073	1.892
		(0.325)	(0.354)	(0.215)	(0.148)	(0.153)	(0.124)	(0.095)	(0.096)	(0.087)

Results for 1000 Monte Carlo replications; standard deviations in parentheses. In each period, the number of observation is 100. $x_{it} \sim U[-1,1]$ . $\beta _1= 2$ and $\alpha _i = \sqrt{T} {\bar{x}}_i + N(0,1)$. Overdispersion factor $\frac{k-1}{\phi +1 }$ represents dispersion degree of variance. Data for overdispersion degree 0 are generated from the binomial model, and data with positive dispersion degree are generated by a beta-binomial DGP

## Appendix B

See Table 7.

**Table 7:** Simulation results $N=500$

$N=500$	Overdispersion (%)	$T=2$			$T=5$			$T=10$		
		Blogit CML	Blogit DV	Pooled logit	Blogit CML	Blogit DV	Pooled logit	Blogit CML	Blogit DV	Pooled logit
$K=2$	0	2.013	2.826	2.234	2.002	2.278	1.987	2.002	2.130	1.870
		(0.170)	(0.254)	(0.112)	(0.082)	(0.097)	(0.068)	(0.053)	(0.057)	(0.046)
	10	2.078	2.936	2.214	2.026	2.307	1.975	2.011	2.139	1.870
		(0.195)	(0.293)	(0.120)	(0.084)	(0.099)	(0.070)	(0.058)	(0.062)	(0.050)
	50	2.361	3.424	1.849	2.142	2.438	1.881	2.062	2.191	1.926
		(0.311)	(0.489)	(0.175)	(0.151)	(0.180)	(0.120)	(0.102)	(0.110)	(0.092)
$K=5$	0	2.003	2.268	2.232	2.001	2.102	1.986	2.000	2.049	1.867
		(0.102)	(0.119)	(0.078)	(0.051)	(0.054)	(0.050)	(0.033)	(0.034)	(0.033)
	10	2.032	2.302	2.237	2.007	2.109	1.984	2.006	2.055	1.868
		(0.108)	(0.126)	(0.088)	(0.052)	(0.055)	(0.051)	(0.035)	(0.036)	(0.034)
	50	2.143	2.435	2.204	2.052	2.157	1.973	2.023	2.073	1.871
		(0.132)	(0.156)	(0.090)	(0.063)	(0.068)	(0.055)	(0.041)	(0.043)	(0.040)
	100	2.289	2.617	2.121	2.103	2.211	1.951	2.050	2.100	1.895
		(0.168)	(0.203)	(0.107)	(0.080)	(0.085)	(0.063)	(0.049)	(0.051)	(0.046)
	200	2.619	3.034	1.852	2.225	2.339	1.882	2.105	2.156	1.932
		(0.292)	(0.363)	(0.152)	(0.137)	(0.146)	(0.109)	(0.093)	(0.095)	(0.083)
$K=10$	0	1.997	2.121	2.235	1.999	2.049	1.984	2.000	2.024	1.867
		(0.071)	(0.076)	(0.067)	(0.034)	(0.035)	(0.042)	(0.023)	(0.023)	(0.029)
	10	2.014	2.140	2.233	2.003	2.053	1.986	2.002	2.026	1.869
		(0.075)	(0.081)	(0.073)	(0.038)	(0.039)	(0.043)	(0.025)	(0.025)	(0.031)
	50	2.066	2.195	2.228	2.023	2.073	1.980	2.013	2.037	1.868
		(0.092)	(0.099)	(0.076)	(0.043)	(0.044)	(0.044)	(0.029)	(0.029)	(0.033)
	100	2.134	2.269	2.206	2.050	2.100	1.974	2.024	2.048	1.871
		(0.108)	(0.117)	(0.083)	(0.050)	(0.051)	(0.048)	(0.035)	(0.035)	(0.035)
	200	2.285	2.443	2.136	2.105	2.157	1.959	2.049	2.074	1.890
		(0.143)	(0.156)	(0.095)	(0.066)	(0.068)	(0.057)	(0.043)	(0.043)	( 0.040)

see “Appendix A”

## References

[CR1] Abrevaya J (1997). The equivalence of two estimators of the fixed-effects logit model. Econ Lett.

[CR2] Barmby T, Nolan M, Winkelmann R (2001). Contracted workdays and absence. Manchester Sch.

[CR3] BfS Bundesamt für Statistik (2016) Arbeitsmarktindikatoren der Schweiz. Neuchâtel, Switzerland

[CR4] Chamberlain G (1980). Analysis of covariance with qualitative data. Rev Econ Stud.

[CR5] Dean CB (1992). Testing for overdispersion in Poisson and binomial regression models. J Am Stat Assoc.

[CR6] Gourieroux C, Monfort A, Trognon A (1984). Pseudo maximum likelihood methods: theory. Econometrica.

[CR7] Kemp G, Santos Silva J (2016) Partial effects in fixed-effects models, United Kingdom Stata Users’ Group Meetings 2016

[CR8] Kunz J, Staub K, Winkelmann R (2018) Predicting fixed effects in panel probit models. York Health, Econometrics and Data Group Working Paper #18/23

[CR9] Machado MP (2004). A consistent estimator for the binomial distribution in the presence of incidental parameters: an application to patent data. J Econom.

[CR10] Maddala GS (1983). Limited-dependent and qualitative variables in econometrics.

[CR11] Mroz TA (1987). The sensitiviy of an empirical model of married women’s hours of work to economic and statistical assumptions. Econometrica.

[CR12] Pagan A, Ullah A (1999). Nonparametric econometrics.

[CR13] Papke LE, Wooldridge JM (1996). Econometric methods for fractional response variables with an application to 401(k) plan participation rates. J Appl Econom.

[CR14] Papke LE, Wooldridge JM (2008). Panel data methods for fractional response variables with an application to test pass rates. J Econom.

[CR15] Ramalho EA, Ramalho JJS, Coelho LMS (2016). Exponential regression of fractional-response fixed-effects models with an application to firm capital structure. J Econom Methods.

[CR16] Steinhauer A (2018) Working moms, childlessness, and female identity. CEPR Discussion Paper Nr. 12929

[CR17] Wooldridge JM (2002). Econometric analysis of cross section and panel data.

